# Ursodeoxycholic acid for the prevention of symptomatic gallstone disease after bariatric surgery: study protocol for a randomized controlled trial (UPGRADE trial)

**DOI:** 10.1186/s12876-017-0674-x

**Published:** 2017-12-20

**Authors:** Thomas C. C. Boerlage, Sylke Haal, L. Maurits de Brauw, Yair I. Z. Acherman, Sjoerd Bruin, Arnold W. J. M. van de Laar, Daan E. Moes, Bart A. van Wagensveld, Claire E. E. de Vries, Ruben van Veen, Ruben Schouten, Marcel G. Dijkgraaf, Paul Fockens, Victor E. A. Gerdes, Rogier P. Voermans

**Affiliations:** 1 Department of internal medicine, MC Slotervaart, Amsterdam, the Netherlands; 2 Department of gastroenterology & hepatology, Academic Medical Center, University of Amsterdam, Meibergdreef 9, 1105 AZ Amsterdam, the Netherlands; 3 Department of surgery, MC Slotervaart, Amsterdam, the Netherlands; 4 Department of surgery, OLVG, Amsterdam, the Netherlands; 5 Department of surgery, MC Zuiderzee, Lelystad, the Netherlands; 6 Clinical Research Unit, Academic Medical Center, University of Amsterdam, Amsterdam, the Netherlands; 7 Department of vascular medicine, Academic Medical Center, University of Amsterdam, Amsterdam, the Netherlands; 8 Department of gastroenterology, MC Slotervaart, Meibergdreef 9, 1105 AZ Amsterdam, the Netherlands

**Keywords:** Gallstones, Bariatric surgery, Roux-en-Y gastric bypass, Sleeve gastrectomy, Ursodeoxycholic acid, Cost-benefit analysis, Randomized controlled trial

## Abstract

**Background:**

The number of bariatric interventions for morbid obesity is increasing worldwide. Rapid weight loss is a major risk factor for gallstone development. Approximately 11 % of patients who underwent Roux-en-Y gastric bypass develop symptomatic gallstone disease. Gallstone disease can lead to severe complications and often requires hospitalization and surgery. Ursodeoxycholic acid (UDCA) prevents the formation of gallstones after bariatric surgery. However, randomized controlled trials with symptomatic gallstone disease as primary endpoint have not been conducted. Currently, major guidelines make no definite statement about postoperative UDCA prophylaxis and most bariatric centers do not prescribe UDCA.

**Methods:**

A randomized, placebo-controlled, double-blind multicenter trial will be performed for which 980 patients will be included. The study population consists of consecutive patients scheduled to undergo Roux-en-Y gastric bypass or sleeve gastrectomy in three bariatric centers in the Netherlands. Patients will undergo a preoperative ultrasound and randomization will be stratified for pre-existing gallstones and for type of surgery. The intervention group will receive UDCA 900 mg once daily for six months. The placebo group will receive similar-looking placebo tablets. The primary endpoint is symptomatic gallstone disease after 24 months, defined as admission or hospital visit for symptomatic gallstone disease. Secondary endpoints consist of the development of gallstones on ultrasound at 24 months, number of cholecystectomies, side-effects of UDCA and quality of life. Furthermore, cost-effectiveness, cost-utility and budget impact analyses will be performed.

**Discussion:**

The UPGRADE trial will answer the question whether UDCA reduces the incidence of symptomatic gallstone disease after Roux-en-Y gastric bypass or sleeve gastrectomy. Furthermore it will determine if treatment with UDCA is cost-effective.

**Trial registration:**

Netherlands Trial Register (trialregister.nl) 6135. Date registered: 21-Nov-2016.

## Background

The number of bariatric interventions for morbid obesity is increasing worldwide. The laparoscopic Roux-en-Y gastric bypass (RYGB) is the bariatric intervention performed most often, although the sleeve gastrectomy is gaining popularity [1]. Rapid weight loss after bariatric surgery is a major risk factor for the development of gallstones [2–4]. Two population-based studies showed that patients who underwent bariatric surgery have a 5.5-fold increased risk of undergoing a cholecystectomy when compared to the general population. The incidence is highest between 7 and 24 months after bariatric surgery [2, 5]. Overall, 8–15% of patients with an intact gallbladder undergoing bariatric surgery will develop symptomatic gallstone disease within two years after surgery [6–10]. The increased risk of gallstone development involves several determinants. Rapid weight loss leads to a change in cholesterol metabolism and consequently increases the concentration of cholesterol in the bile to a level at which not all cholesterol can be dissolved by the bile salts. The undissolved cholesterol is prone to crystallize into stones, especially in the presence of calcium and mucin, a glycoprotein that stimulates cholesterol crystal aggregation [3, 11]. The concentration of mucin in the bile increases 10–20 fold after bariatric surgery. The exact mechanism behind this increase is unknown [11]. The risk of gallstone formation is also increased by incomplete and slower emptying of the gallbladder, causing stasis of bile [3]. Symptomatic gallstone disease can lead to biliary colics, and severe complications such as cholecystitis, cholangitis and pancreatitis. The risk of acute (biliary) pancreatitis is 50-fold increased in patients who underwent bariatric surgery, compared to the general population [12]. In case of cholangitis, biliary pancreatitis or pain due to symptomatic bile duct stones, conventional endoscopic retrograde cholangiopancreatography (ERCP) cannot be performed due to the altered anatomy after RYGB. Therefore more invasive procedures, such as an ERCP via double balloon enteroscopy or a surgically created gastrostomy, or percutaneous transhepatic drainage need to be performed in these patients [13]. Most patients with gallstone disease have milder disease and only suffer from biliary colics. These patients are treated with a laparoscopic cholecystectomy, which requires hospital admission and can be difficult due to adhesions caused by the previous bariatric surgery. In general, the chance of conversion from laparoscopic to open cholecystectomy is up to three times higher after previous abdominal surgery [14, 15]. Conversion to open cholecystectomy increases the risk of postoperative complications and the hospital costs. [16] Another severe complication of cholecystectomy is bile leak due to bile duct injury [17, 18].

Several strategies have been proposed for the prevention of gallstone disease in patients undergoing bariatric surgery. Although some authors advocate routine cholecystectomy, this prolongs the duration of surgery and admission, increases the number of laparoscopy incisions required for surgery and carries a risk for complications, especially in this morbidly obese population [19–21]. A selective approach in which all patients undergo pre-operative ultrasound and those with stones in the gallbladder undergo concomitant cholecystectomy, has been proven to lead to a higher morbidity and is therefore neither recommended [20, 22]. A patient-based approach in which only patients at high risk of developing gallstone disease undergo treatment is not possible, as studies have failed to identify specific risk groups in the bariatric population at whom prophylactic treatment could be directed [23–25]. This is because the risk of gallstone development is very strongly correlated with the amount of weight loss [24, 26]. The amount of weight loss varies per patient and cannot be predicted beforehand. Other patient characteristics such as the traditional risk factors for gallstone formation play a minor role in this specific population.

An opportunity to medically prevent symptomatic gallstone disease during rapid weight loss is the administration of ursodeoxycholic acid (UDCA). UDCA is an orally taken bile acid that is known to prevent the formation of gallstones by increasing bile flow and reducing its lithogenicity. It is well tolerated with few side effects. The most prevalent side effect is diarrhoea in 2–9% of patients [27].

Five randomized controlled trials have studied the use of UDCA for gallstone prophylaxis after bariatric surgery (RYGB, vertical banded gastroplasty or adjustable gastric banding), the data of which has been pooled in two meta-analyses [28, 29]. In summary, UDCA for 3 to 6 months effectively prevents the formation of gallstones up to 24 months after bariatric surgery. The relative risk in an intention-to-treat analysis was 0.43 (0.22–0.83) in favour of UDCA [29]. One recent trial studying the effect of UDCA after sleeve gastrectomy showed similar results [30]. However, the primary endpoint of all studies consisted of the formation of gallstones on ultrasound and not symptoms of, or medical interventions for gallstones. These trials do therefore not provide definite evidence regarding the use of UDCA, as 60–80% of patients with gallstones will remain asymptomatic [4, 8, 31]. Apart from the absence of a clinically relevant primary endpoint, most studies were underpowered and showed a high loss to follow-up. Finally, most trials included different types of bariatric surgery, which lead to less weight loss in comparison with RYGB. Because the amount of weight loss is correlated with the risk of symptomatic gallstone disease, these studies probably even underestimate the positive effect of UDCA. The current uncertainty about the use of postoperative gallstone prophylaxis is reflected in the different guidelines. The 2013 guideline by the American Society of Metabolic and Bariatric Surgeons states that both prophylactic cholecystectomy and the postoperative use of ursodeoxycholic acid may be considered, but makes no definitive statement about either of the preventive strategies [32]. This study is designed to provide evidence regarding the prophylactic use of UDCA in preventing symptomatic gallstone disease postoperatively.

## Methods/design

### Study population

**Fig. 1:**
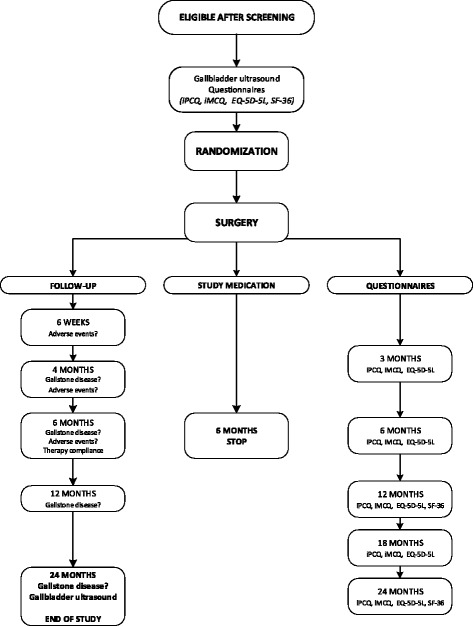
Study flowchart

**Fig. 2:**
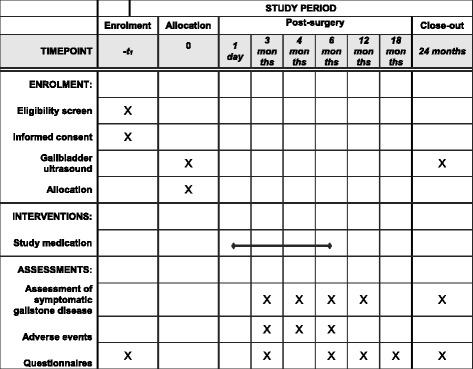
Template for the schedule of enrolment, interventions, and assessments

We will conduct a multicenter, randomized, placebo-controlled, double-blind study comparing the prophylactic use of UDCA versus placebo in patients undergoing RYGB or sleeve gastrectomy. (Figs. 1 and 2) The study population will consist of patients scheduled to undergo RYGB or sleeve gastrectomy in three high-volume bariatric centers in the Netherlands. Patients are first informed about the study during the preoperative screening program. If patients are considered eligible for bariatric surgery after multidisciplinary consultation, they are screened for the study and asked for informed consent. Patients with prior bariatric or gallbladder surgery will be excluded. Approximately 13–20% of patients undergoing bariatric surgery already have asymptomatic gallstones [6, 19, 31]. Previous trials excluded these patients, but patients who already have gallstones might not have a higher risk of becoming symptomatic after bariatric surgery [23]. In current practice, these patients receive no extra treatment or prophylaxis after bariatric surgery. Therefore, these patients will be included in this trial as well.

### In- and exclusion criteria

#### Inclusion criteria

In order to be eligible to participate in this study, a subject must meet all of the following criteria:

∙ Scheduled to undergo Roux-en-Y gastric bypass or sleeve gastrectomy for morbid obesity

∙ An intact gallbladder

#### Exclusion criteria

A potential subject who meets any of the following criteria will be excluded from participation in this study:

∙ Symptomatic gallstone disease already present before RYGB

∙ Prior bariatric surgery

∙ Prior gallbladder surgery

∙ Ascertained or presumptive hypersensitivity to active or excipient ingredients of UDCA.

∙ Inflammatory bowel disease and other conditions of the small intestine and liver which may interfere with enterohepatic circulation of bile salts (ileal resection and stoma, extra and intra-hepatic cholestasis, severe liver disease)

∙ Intake of investigational drug within the last 30 days before the screening

### Primary outcome measure

The primary endpoint of this study is symptomatic gallstone disease after 24 months, defined as hospital admission or hospital visit for symptomatic gallstone disease. Hospital visit is a condition, because all patients with noteworthy symptoms will eventually visit the hospital. Mild and self-limiting complaints are not a large burden to the health care system or to the patient, and usually gallstone involvement is not objectified in these patients. Symptomatic gallstone disease is defined as biliary pancreatitis, acute cholecystitis, choledocholithiasis, cholangitis, or biliary colics. Acute pancreatitis is diagnosed in the presence of two of the three following: upper abdominal pain; serum lipase or amylase levels above 3 times the upper level of normal; characteristic findings of acute pancreatitis on cross-sectional abdominal imaging [33]. Pancreatitis is regarded of biliary origin when imaging reveals gallstones, sludge or a dilated common bile duct, or when laboratory investigation reveals an alanine aminotransferase (ALT) level > 2 times higher than normal values, with ALT > aspartate aminotransferase (AST). Acute cholecystitis and cholangitis are diagnosed according to the diagnostic criteria of the updated Tokyo Guidelines [34]. Acute cholecystitis is diagnosed in the presence of at least one local sign of inflammation (either Murphy’s sign or right upper quadrant mass/pain/tenderness) and at least one systemic sign of inflammation (fever, elevated C-reactive protein, or elevated white blood cell count). Choledocholithiasis is defined as the presence of stones in the extrahepatic bile ducts as proven by clinical imaging, or clinical suspicion based on abnormal liver function tests in combination with upper abdominal pain for which an ERCP or PTC was indicated. Cholangitis is diagnosed when there is a sign of systemic inflammation (fever/shaking chills or laboratory evidence of inflammatory response) and either clinical or laboratory evidence of cholestasis (total bilirubin ≥34 μmol/L, or increased serum alkaline phosphatase, gamma-glutamyl transpeptidase, ALT, or AST >1.5× higher than the upper limit of normal value) or evidence of cholestasis on biliary imaging (biliary dilatation or evidence of the etiology on imaging). Biliary colics are defined as upper abdominal pain (either right upper quadrant or epigastric pain) lasting at least 30 min with gallstones visible on ultrasound, according to the Rome criteria [35]. In case of doubt whether a participant has reached the primary endpoint, endpoint adjudication will be done by an independent blinded committee.

### Secondary outcome measure

Secondary endpoints consist of:

1. The presence of gallstones on ultrasound at 24 months
2. The number of cholecystectomies in both groups
3. Side-effects of UDCA
4. Therapy compliance
5. Quality of life, cost-effectiveness, cost-utility and budget impact

#### Quality of life, cost-effectiveness, cost-utility and budget impact analyses

Participating patients are monitored regarding use of health care, quality of life (SF-36) and health utility (EQ5D-5 L), productivity loss and out-of-pocket expenses. The primary outcomes of these analyses will be the costs per patient without poor outcome (defined as symptomatic gallstone disease), and costs per quality adjusted life year (QALY). A budget impact analysis from a governmental and health insurer perspective will be performed, describing the financial consequences of prophylactic use of UDCA and reduced numbers of surgical interventions for the extramural medication budget and budget for specialized health care respectively.

### Power calculation

The prevalence of symptomatic gallstone disease after bariatric surgery is 11% in the eligible population. [10] It is estimated that UDCA gives a 2- to 3-fold decrease in gallstone development when compared to placebo [28, 29]. We decided to calculate the power based on a 2-fold reduction in gallstone disease, to minimize the risk of an underpowered study. Assuming a 50% reduction in symptomatic gallstone disease from 11 to 5.5%, a 2-sided 5% alpha, power of 80%, and 20% dropout, 980 patients in total are needed (chi square test without correction for continuity).

### Randomization

Before randomization an ultrasound of the gallbladder will be performed in all patients. Patients are blinded for the outcome of the ultrasound, except in case of incidental findings that require medical attention. Patients are then randomized to receive either UDCA 900 mg once daily or placebo in a 1:1 ratio. Randomization is performed in blocks and stratified for the presence of cholecystolithiasis and the type of surgery. It was decided to stratify because it is uncertain whether these patients have the same risk of developing symptomatic gallstone disease as patients without pre-existing gallstones. An unequal distribution of these patients between the treatment groups might negatively affect the power of the study. Randomization is performed using a computerized randomization program (ALEA), which is validated for use in randomized clinical trials. The block size randomly varies between 4, 6 and 8.

### Study medication

UDCA will be prescribed as 900 mg once daily. This dose was shown to be more effective in preventing gallstone formation than 300 mg once daily [29]. The placebo tablets are similar-looking and the placebo group is treated according to the same treatment schedule as the intervention group. The study medication is started within two weeks after surgery. Treatment duration is 6 months, because it is expected that UDCA use for longer than 6 months has no extra benefit. The risk of developing new gallstones is maximal in the period of rapid weight loss and decreases when the weight stabilizes. Seventy-five percent of the total weight loss resulting from RYGB, is lost in the first 6 months. After these first 6 months, the weight loss decreases and eventually stops at 18–24 months after surgery [36]. Therefore the window of opportunity in preventing gallstone formation exists in the first 6 months after surgery. Less than 5% of the patients who have not formed gallstones at 6 months, will have developed gallstones at 12 or 18 months after surgery [4]. When the rapid weight loss stops, gallstones may even dissolve spontaneously in time [37]. In a retrospective study, 6 or 12 months of UDCA use made no difference in the preventive effect [38].

### Follow-up

The follow-up duration will be 24 months. Newly formed gallstones will typically become symptomatic in the first 6–18 months after formation. The mean time from surgery to the development of symptomatic gallstone disease is 11 months [7, 24]. The longer gallstones remain asymptomatic, the smaller the chance that they will ever become symptomatic [39]. A prospective cohort study showed that in all patients who developed symptomatic gallstone disease, symptoms occurred in the first 29 months after surgery. None of the remaining patients underwent cholecystectomy in a follow-up period up to 144 months after bariatric surgery. Therefore, a follow-up period longer than 24 months is not expected to result in a significantly higher rate of symptomatic gallstone disease [7].

At the follow-up visits during the first 6 months, symptomatic gallstone disease, side effects of UDCA and other possibly related (serious) adverse events are assessed. Therapy compliance is measured by asking the patient to indicate the average number of days (0–7) the medication was taken. Pill count is performed after 6 months to objectify the therapy compliance [40]. Hereafter, patients are asked for the occurrence of symptomatic gallstone disease at each follow-up visit. At the 24-months visit, the gallbladder ultrasound is repeated. Furthermore, patients fill in the EQ5D-5 L, iMTA Medical Consumption Questionnaire (iMCQ) and iMTA Productivity Costs Questionnaire (iPCQ) preoperatively and at 3, 6, 12, 18 and 24 months. Furthermore, the SF-36 is already administered in regular care preoperatively and at 12 and 24 months. The iPCQ and iMCQ have been slightly adjusted for this study in order to be more specific for the study population and procedure. In the original validated version of the iMCQ and iPCQ the authors explicitly permitted these kind of changes to the questionnaires without limiting the validity [41].

### Adverse events

UDCA is a drug with little known side effects. Patients treated with UDCA experienced diarrhoea in several studies, but it is still unclear whether this can be attributed to UDCA or to the underlying disease [27]. A meta-analysis of UDCA for primary biliary cirrhosis showed no difference in the prevalence of adverse events between the UDCA group and controls [42]. Skin reactions are known from case reports, but have not been described in large series [27].

In regular care, postoperative diarrhoea is treated with generous intake of fluids and antidiarrhoeal drugs when necessary. In case of diarrhoea (defined according to the World Health Organization as the passage of 3 or more loose or liquid stools per day) that is considered burdensome by the patient, does not respond to regular treatment, persists for longer than one week and is possibly related to the study drug, the study drug dose can be halved to 450 mg once daily. If the diarrhoea still persists after one week of follow-up, the study drug can be discontinued.

### Data handling and analysis

All data will be stored in an electronic case record form, which was designed in the program Castor Electronic Data Capture (Ciwit BV, the Netherlands). This software is compliant with Good Clinical Practice.

The primary analysis will be on an intention-to-treat basis, including all randomized patients. The difference in the prevalence of symptomatic gallstone disease between the study groups will be compared using the chi-square test. To adjust for the stratification for pre-existent gallstones, logistic regression will also be performed. For missing data a multiple imputation approach will be selected (and justified) that best fits the observed missing data pattern at the time of analysis. A sensitivity analysis will be performed for which only cases with complete follow-up will be analyzed.

For the cost-effectivity and cost-utility analysis, incremental cost-effectiveness ratios will be calculated as the extra costs per additional patient without symptomatic gallstone disease and as the extra costs per QALY gained. Medical, patient and employer costs will be included in the evaluation. The medical costs cover the costs of diagnosis, admission and treatment for symptomatic gallstone disease. The patient costs include the expenses for over-the-counter medication, non-reimbursable dietaries, and health care related travel. The employer costs reflect losses of productivity resulting from absenteism and presenteism. The mid-term budget impact (up to four calendar years) of standard prescription of UDCA after bariatric surgery will be assessed from governmental, insurer and hospital care provider perspectives, in accordance with the recent guideline [43]. If >10% of all included patients received sleeve gastrectomy, then an exploratory subgroup analysis of differences in QALYs and costs will be performed to assess the need for extrapolation scenarios that account for the potential future growth in popularity of SG among patients.

### Study integrity

A grant for his study was obtained from ZonMW (The Netherlands Organisation for Health Research and Development). The protocol for this study was peer-reviewed by external reviewers in the course of this grant application. The study protocol was reviewed and approved by the medical research ethics committee of the MC Slotervaart / Reade, and authorized by the competent authority in accordance with the Dutch Medical Research Involving Human Subjects Act (WMO).

### Study monitoring and safety

Monitoring will be done by an independent monitor. Because the study was classified as very low risk, monitoring will consist of an initiation visit to all participating sites, and an annual visit to each site after initiation. The monitor will focus on the quality of data collection for the primary endpoint and patient safety. Furthermore, there is an independent reviewer regarding drug safety. When 50% of the total number of patients has finished the six months of UDCA, the independent reviewer will be informed about all serious adverse events in both study groups, without deblinding. Due to the design of the study an interim analysis for effect or futility is not feasible. Patients will develop symptomatic gallstone disease at a mean time of 11 months after surgery. As inclusion is scheduled to take approximately 12 months, an interim analysis will have no consequences for the number of patients that has to be included.

## Discussion

This will be the first randomized controlled trial to study the effectivity of UDCA for the prevention of gallstones after bariatric surgery with a clinically relevant endpoint. This study will also provide insight in the effectiveness and cost-effectiveness of this intervention.

This study will include both patients undergoing RYGB and sleeve gastrectomy, although the majority of patients are expected to undergo RYGB. The sleeve gastrectomy is increasingly being performed worldwide and in some countries the sleeve gastrectomy is performed more often than the RYGB. However, in most countries, including the Netherlands, the RYGB is still the most frequently performed intervention. In the participating centers, approximately 10% of bariatric interventions have been sleeve gastrectomies over the past years, and this number is increasing slowly. Previous studies indicate that the risk of gallstone disease and the effect of UDCA are similar after RYGB and sleeve gastrectomy [30, 44]. Therefore, the inclusion of both patients undergoing RYGB and sleeve gastrectomy is not expected to introduce bias, and will extend the validity of this study for clinical practice.

Loss to follow-up is expected to be low, because the 2-year follow-up rate is already 95% in regular care [10, 45]. However, it was decided to implement a 20% correction for drop-out to minimize the risk of an underpowered study. This was also done because therapy incompliance has been an important factor in previous studies with UDCA. To objectify the influence of therapy incompliance in this study, a pill count will be performed.

In conclusion, this randomized controlled trial is designed to provide evidence regarding the effectiveness of UDCA for the prevention of symptomatic gallstone disease after bariatric surgery. The main advantage over previous studies is the presence of a clinically relevant endpoint, namely symptomatic gallstone disease, and the follow-up duration of 24 months.

## Abbreviations

ALT: alanine aminotransferase
AST: aspartate aminotransferase
ERCP: endoscopic retrograde cholangiopancreatography
iMCQ: iMTA Medical Consumption Questionnaire
iPCQ: iMTA Productivity Costs Questionnaire
QALY: quality adjusted life year
RYGB: Roux-en-Y gastric bypass
UDCA: ursodeoxycholic acid
